# The impact of online food delivery applications on dietary pattern disruption in the Arab region

**DOI:** 10.3389/fpubh.2025.1569945

**Published:** 2025-06-10

**Authors:** Radwan Qasrawi, Suliman Thwib, Ghada Issa, Malak Amro, Razan AbuGhoush, Maha Hoteit, Sahar Khairy, Narmeen Jamal Al-Awwad, Khlood Bookari, Sabika Allehdan, Dalal Alkazemi, Haleama Al Sabbah, Salima Al Maamari, Asma H. Malkawi, Reema Tayyem

**Affiliations:** ^1^Department of Computer Science, Al-Quds University, Jerusalem, Palestine; ^2^Department of Computer Engineering, Istinye University, Istanbul, Türkiye; ^3^Food Sciences Unit, National Council for Scientific Research of Lebanon (CNRS-L), Beirut, Lebanon; ^4^PHENOL Research Program, Faculty of Public Health, Section 1, Lebanese University, Beirut, Lebanon; ^5^National Nutrition Institute, Cairo, Egypt; ^6^Department of Clinical Nutrition and Dietetics, Faculty of Applied Medical Sciences, The Hashemite University, Zarqa, Jordan; ^7^Department of Nutrition and Integrative Health, Faculty of Allied Medical Sciences, Middle East University, Amman, Jordan; ^8^Clinical Nutrition Development, Applied Medical Science College, Taibah University, Medina, Saudi Arabia; ^9^Department of Biology, College of Science, University of Bahrain, Zallaq, Bahrain; ^10^Department of Food Science and Nutrition, College of Life Sciences, Kuwait University, Kuwait City, Kuwait; ^11^Department of Public Health, College of Health Sciences, Abu Dhabi University, Abu Dhabi, United Arab Emirates; ^12^Nutrition Department, Ministry of Health, Muscat, Oman; ^13^Ibn Khaldon Center for Humanities and Social Sciences, Qatar University, Doha, Qatar; ^14^Department of Nutrition Sciences, College of Health Sciences, Qatar University, Doha, Qatar

**Keywords:** food delivery applications, online food delivery, dietary patterns, machine learning, dietary disruptions

## Abstract

**Background:**

While online food delivery applications (OFDAs) offer convenient food accessibility, their impact on dietary behaviors remains insufficiently explored, especially in the Arab region. This study applies machine learning (ML) techniques to identify the key behavioral and nutritional factors contributing to dietary disruption linked to OFD platforms.

**Methods:**

We conducted a cross-sectional study which involved 7,370 adults across 10 Arab countries using a comprehensive online survey. The study employed an ensemble ML approach, comparing Random Forest, XGBoost, CatBoost, and LightGBM tree-based models to analyze 31 features across six domains: demographics, ordering frequency, food preferences, nutritional perceptions, behavioral factors, and service attributes. Model performance was evaluated using multiple metrics, including sensitivity, precision, F1-score, and AUC. Clear interpretation of the risk factors was explained using partial dependence plots.

**Results:**

The findings revealed that the strongest predictors of dietary disruption were excessive food consumption, altered meal routines, and preferences for fatty foods. Younger individuals, males, and those with higher BMI reported higher disruption rates. Lebanon and Bahrain showed the highest rates for notable disruption, while Oman reported the lowest. ML analysis demonstrated high predictive performance, with Random Forest achieving the highest sensitivity (94.3%) and F1-score (89.3%). Feature importance analysis identified behavioral factors as more influential than socioeconomic indicators.

**Conclusion:**

OFDAs offer valuable convenience and market expansion while simultaneously posing significant challenges to maintaining optimal dietary health. With strategic interventions and public health collaborations, these platforms can shift from being disruptors of healthy dietary habits to catalysts for improved nutrition and well-being in the Arab region and beyond.

## Highlights

- Young adults (18–30 years) are a high-risk group for experiencing dietary disruption through OFDAs.
- Behavioral factors, particularly excessive consumption, disrupted meal routines, and preference for fatty foods are the strongest predictors of dietary disruption.
- Healthier foods have a protective effect.

## 1 Introduction

Online food delivery applications (OFDAs) have fundamentally transformed food consumption patterns across the globe. This digital shift has introduced a significant change in food accessibility and eating behaviors, enabling consumers to access diverse food options with minimal effort (1). For instance, algorithm-driven personalization and target marketing strategies have been shown to influence food choices (2, 3). The widespread adoption of OFDAs in the Arab region is largely driven by growing internet utilization, urbanization, and increasingly hectic lifestyles (4).

In the Arab region, popular OFD platforms such as Talabat, Deliveroo, and Uber Eats have achieved significant market penetration. A recent survey indicates that Talabat was the most popular choice, likely due to its widespread and performance in the region (5).

While these platforms promise convenience, they also raise concerns about potential disruptions to traditional dietary habits and broader implications for public health (6). These disruptions are particularly noticeable in regions where cultural and traditional diets are integral to identity and well-being, such as the Arab world.

A multitude of studies suggests that the benefits of OFDAs can be circumstantial. For example, during COVID-19, they were notably instrumental in allowing people to source their food without leaving their homes (7). OFDAs can also increase food availability and variety while lowering cost and effort barriers to consumption. They may even offer healthier options that consumers might not otherwise consider. However, studies indicate that their negative effects outweigh the benefits, challenging public health systems as they promote sedentary lifestyles and facilitate access to cheap, unhealthy options (7, 8).

The impact of OFDAs on dietary patterns has been a subject of growing concern. Research indicates a trend toward increased consumption of calorie-dense and nutrient-poor meals, with a concomitant decline in the intake of home-cooked and culturally significant foods (6, 7, 9, 10). The availability of unhealthy foods can play a role in broader health issues, including rising obesity rates, cardiovascular diseases, and diabetes (3, 4). Studies reveal how OFDA usage is linked to adverse health outcomes, emphasizing the urgency of examining these relationships in local contexts (11). OFDAs encourage dependency on processed and fast foods, which often lack essential nutrients while promoting excessive calorie intake (1, 12, 13). The implications are particularly explicit in urbanized regions where reliance on these platforms is highest. In the Arab region, dietary habits have traditionally revolved around whole grains, legumes, fruits, and lean proteins—an aspect of the Mediterranean diet often lauded for its health benefits (14). However, globalization and urbanization have introduced significant dietary shifts, including increased consumption of fast foods, sugary beverages, and processed meals (11, 15, 16). OFDAs exacerbate this trend by offering accessibility and convenience that poorly aligns with traditional dietary practices.

While some studies touch upon these dietary shifts, there remains a shortage in research focusing explicitly on the role of OFDAs in driving these changes within the Arab context (17, 18). Furthermore, behavioral determinants, including personal food preferences and the broader influence of societal trends and digital marketing strategies, play a pivotal role in shaping food choices, particularly in the context of OFDAs. Factors such as time constraints, marketing influences, and perceived convenience drive consumers toward quick, yet consequentially less healthy options, promoting fast food consumption. Several studies highlighted how marketing strategies, such as gamification and personalized recommendations, significantly impact younger demographics, normalizing the consumption of less nutritious food (16, 19–21). For example, (22) shows that people tend to evaluate the price of their food rather than the calorie count when making dietary choices. This promotes the need to explore how OFDAs affect consumer behavior, especially among populations already predisposed to lifestyle-related health issues.

Recent studies have increasingly focused on the role of food delivery applications in shaping dietary habits and public health outcomes. One study conducted in Saudi Arabia found that extensive use of these platforms was strongly associated with increased fast-food consumption and potentially unhealthy eating behaviors, highlighting the convenience-driven shift in dietary patterns (23). Research in the United States has documented how the rapid expansion of digital food ordering correlates with higher caloric intake and an elevated risk of obesity, highlighting broader negative health implications (8). Complementing these findings, a recent investigation in Jordan revealed that while online food delivery apps provide a diverse range of food choices, factors such as food appearance, price, and delivery speed predominantly drive consumer preferences, often at the expense of healthy food selections (13). Together, these studies contribute to a deeper understanding of how digital food environments are influencing consumer behavior and health outcomes across different regions.

Machine learning (ML) offers promising tools for analyzing the complex relationships between OFDA usage, behavioral determinants, and nutritional outcomes. Several ML approaches are particularly relevant for examining OFDA effects: supervised learning methods like decision trees and random forests can classify dietary patterns and predict nutritional quality (24); natural language processing techniques can extract dietary patterns from unstructured food-related text data (25); while deep learning techniques enable analysis of food images to estimate nutritional content. These approaches require diverse data types including user demographic information, ordering patterns, and nutritional composition of meals (26). Recent advancements in ML techniques enable researchers to analyze large datasets with high precision. Research studies demonstrated the potential of ML in identifying dietary trends, predicting health risks, and evaluating interventions aimed at improving food choices (9, 27–29). Despite this potential, implementing ML research in the Arab region faces unique challenges, including limited public health data infrastructure, privacy concerns related to cultural sensitivities, and significant regional variation in technological adoption rates (30, 31). Additionally, algorithmic biases may reinforce existing nutritional disparities if not carefully calibrated to local contexts (32). However, ML remains underutilized in examining the localized impacts of OFDAs, particularly in culturally distinct and rapidly urbanizing regions such as the Arab world. Emerging studies in 2023 further highlight the intersection of technology, dietary behavior, and health outcomes. One study shows the role of algorithm-driven personalization in influencing food orders (33), while others examine the nutritional qualities of meals frequently ordered via OFDAs, revealing significant deviations from recommended dietary guidelines (34, 35). These findings provide a foundation for further exploration into how digital platforms impact health and nutrition in the Arab world.

This study addresses these gaps by applying machine learning techniques to analyze behavioral and nutritional determinants of dietary disruption associated with OFDAs in the Arab region. By leveraging advanced analytics, this research seeks to reveal trends, identify vulnerable populations, and provide actionable insights for policymakers and public health practitioners.

## 2 Materials and methods

### 2.1 Study design

Model data was derived from a cross-sectional survey conducted through an online questionnaire from February to June 2024. The survey captured demographic information, the frequency of online food delivery (OFD) application usage, participant attitudes toward OFD, perceived service quality, usage patterns, and preferences for ordering healthy versus unhealthy foods. The survey was created in alignment with relevant literature and reviewed by partner experts from the participating countries to establish face and content validity. It was first drafted in English and then translated into Arabic. The bilingual survey versions were carefully and culturally adapted to ensure semantic equivalence. Since differences in interpretation and linguistic nuances between Arabic and English could lead to variability in participants’ understanding and responses, validation was carried out using forward and backward translation methods, combined with expert panel reviews to confirm conceptual equivalence. The questionnaire contained 120 close-ended questions, including multiple-choice, Likert-scale, dichotomous, and checklist questions.

For validation, four experts in nutrition, public health, and food hygiene reviewed the questionnaire for cultural relevance and clarity. It was revised to align with each country’s context, resolving any disagreements by modifying or removing questions. The final version collected demographic data (age, gender, nationality, residency, education, marital status) and examined food delivery app usage, attitudes, and quality perceptions.

The dataset comprised responses from 7,370 individuals across 10 Arab countries: Bahrain, Egypt, the United Arab Emirates, Jordan, Kuwait, Lebanon, Oman, Palestine, Qatar, and Saudi Arabia. The target population included adults aged 18 to 60 years who were nationals or residents of these countries. Data collection was conducted using a non-probability convenience sampling method, with the goal of gathering between 200 and 400 responses per country. The survey was accessible online at https://bcite.org/food/, and all respondents provided informed consent before participating. Exclusion criteria were applied to those outside the specified age range and those not fluent in either Arabic or English. Recruitment occurred through university networks and social media platforms across the 10 Arab countries. University faculty and researchers shared the survey link with students, staff, and academic networks, while platforms like Facebook, Twitter, and WhatsApp helped reach a broader audience. To reduce selection bias, demographic monitoring ensured balanced representation of age, gender, and nationality, with targeted outreach to underrepresented groups. The use of anonymous responses and bilingual accessibility further minimized bias and enhanced data quality. Several strategies were also implemented to mitigate recall and social desirability bias. Respondents were asked to report recent OFDA usage within short recall periods, such as the past week or month, minimizing recall errors. Confidentiality was assured to encourage honest reporting and reduce social desirability bias. The questions were carefully phrased in neutral language to avoid influencing answers or creating judgmental perceptions. Additionally, validation questions were integrated to cross-check responses and identify potential inconsistencies, improving the overall reliability of the data.

### 2.2 Study features

The study features were categorized into six main domains, where dietary pattern disruption is the target variable. The complete categorization of study features is presented in Table 1.

**Table 1:** Categorization of study features for dietary disruption analysis.

Domain/Category	Features
Target variable	Ordering food via online is associated with disrupted dietary patterns and eating habits
Demographics	Residency (Bahrain, Egypt, Emirates, Jordan, Kuwait, Lebanon, Oman, Palestine, Qatar, and Saudi Arabia), age, gender, education, employment, family income, marital status, BMI.
Ordering frequency	Breakfast orders, lunch orders, dinner orders, snack orders, dessert orders.
Food preferences	Popular fast foods, bakery items, coffee and tea, soft drinks.
Nutritional perceptions	High sugar content, high fat content, high salt content.
Behavioral factors	Trying new cuisines, lack of time to cook, weakened parental control, disrupted meal routines, excessive consumption
Service attributes	Food quality, food taste, affordable prices, access to healthier food, customizable orders.

### 2.3 Data preprocessing

The preprocessing pipeline included data cleaning, feature encoding, normalization, missing value imputation, and class balance adjustment. Initial cleaning involved inspecting survey responses, removing duplicate entries based on respondent IDs, timestamps, and patterns, and consolidating redundant categories.

Categorical variables were encoded using one-hot encoding, which avoids imposing arbitrary ordinal relationships and preserves the categorical nature of the data. Outliers were handled with the Interquartile Range (IQR) method, capping extreme values at 1.5 times the IQR below the first quartile and above the third quartile. Finally, standard scaling was applied to normalize features, ensuring consistency across variables (36).

For handling missing values, a multi-level imputation strategy was implemented. First, Cramér’s V coefficient was employed to identify the strongest correlations between features containing missing values and other columns in the dataset. This measure is particularly suitable for categorical data, as it quantifies the association between two nominal variables, making it ideal for identifying relationships in our dataset. Using these correlations, initial imputation was performed using the grouped mode of the most highly correlated feature for each column with missing values (37). Any remaining missing values were then imputed using k-Nearest Neighbors (k-NN), which leverages the similarity between observations to predict missing values based on neighboring data points, preserving local patterns in the data. This approach is particularly effective when the dataset exhibits strong relationships between features, as it uses the feature space to infer missing values rather than relying on statistical assumptions (38).

Class imbalance in the training set was addressed by implementing a hybrid approach combining One-Sided Selection (OSS) (39) and k-means SMOTE (Synthetic Minority Over-sampling Technique) (40). OSS was applied to clean the majority class by removing redundant and borderline majority instances that might contribute to overfitting. This process retains only the most representative majority class samples while eliminating noise. Following OSS, k-means SMOTE, an enhancement of traditional SMOTE that first clusters the minority class using k-means clustering, was applied. This clustering step ensures that synthetic samples are generated within natural data groupings, thereby maintaining the underlying data distribution and avoiding the creation of unrealistic synthetic samples that could occur with standard SMOTE.

### 2.4 Statistical analysis

Descriptive analyses of demographic frequencies and percentages provided insights into sample composition and distribution, which served as a basis for advanced ML analysis. Univariate analysis using *F*-tests was performed to assess the significance of individual predictors. Associations between predictors and dietary disruption patterns were then examined using multinomial logistic regression, which is ideal for categorical outcomes with multiple levels while accounting for confounders. The model estimated odds ratios (ORs) with 95% confidence intervals (CIs) to quantify associations, with statistical significance set at *p* < 0.05.

### 2.5 ML analysis

An ensemble-based machine learning approach was employed to identify complex, non-linear relationships between behavioral, nutritional, and demographic factors contributing to dietary disruption. Specifically, four tree-based models—Random Forest, CatBoost, XGBoost, and LightGBM—were selected for their ability to handle categorical data and provide interpretable results. These models were chosen over deep learning because the study’s objective was to deliver actionable insights into dietary disruption, which requires models that are both accurate and interpretable. Tree-based ensemble methods offer clear feature importance rankings and allow for the generation of interpretability tools such as partial dependence plots (PDPs) (41). In contrast, deep learning models are often considered “black boxes” due to their complex architectures and lack of transparency.

To optimize the performance of these models, hyperparameter tuning was conducted using the OPTUNA framework, a state-of-the-art tool for Bayesian hyperparameter optimization. Unlike grid search or random search, which inefficiently roam the entire search space, OPTUNA employs a Bayesian optimization approach to intelligently explore the hyperparameter space. It considers the performance of previous hyperparameter combinations to guide the selection of the next set of hyperparameters, significantly reducing the number of evaluations needed to find optimal configurations. This makes OPTUNA highly efficient, especially for large datasets and complex models. The framework also supports parallel processing, enabling faster convergence and reducing computational overhead. Hyperparameters tuned included the number of trees, learning rate, maximum depth, and regularization parameters, among others. OPTUNA’s ability to balance exploration and exploitation, combined with its automatic algorithm selection, ensured that the models were optimized for both performance and generalizability (41).

**Figure 1:**
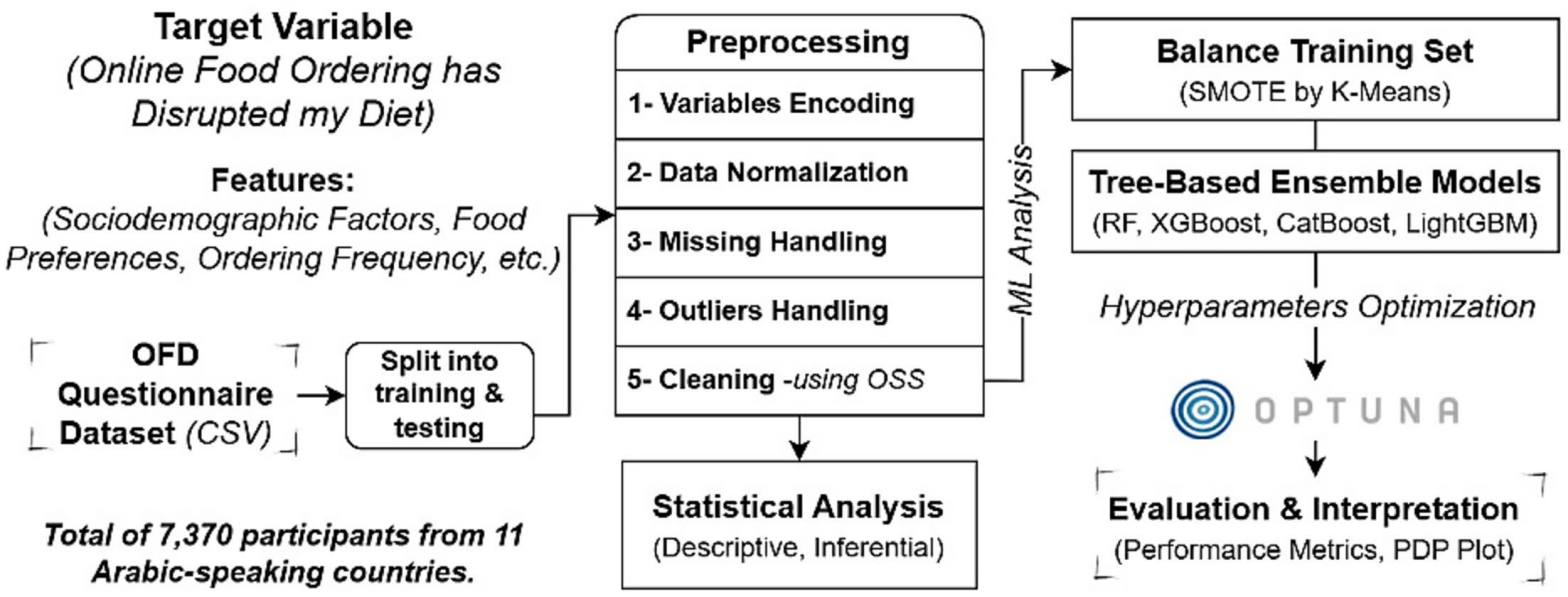
Methodological framework for identifying key predictors of dietary disruption using ensemble machine learning models.

The complete architectural framework of the methodology is illustrated in Figure 1.

### 2.6 Validation and verification

A comprehensive validation and verification process was implemented to ensure the reliability and robustness of the ensemble-based machine learning approach. Model evaluation was performed using 10-fold cross-validation, a robust strategy that ensures the performance metrics (e.g., sensitivity, precision, F1-score, accuracy, AUC) are not biased by a single train-test split. This approach also helps prevent overfitting by averaging performance across multiple data splits, providing a more reliable estimate of the models’ generalizability. Additionally, tree-based ensemble methods incorporate built-in mechanisms to reduce overfitting, such as bagging (in Random Forest) and boosting (in XGBoost, CatBoost, and LightGBM). These techniques ensure that the models generalize well to unseen data, which is critical for reliable predictions.

The OPTUNA framework was used for hyperparameter tuning, systematically exploring the hyperparameter space to identify optimal configurations while avoiding overfitting. The performance of all four models was systematically compared using metrics such as sensitivity, precision, F1-score, accuracy, AUC, and prediction time. Feature importance rankings were extracted from the best-performing model, and partial dependence plots (PDPs) were generated to improve interpretability and validate the direction and magnitude of each predictor’s effect on dietary disruption. PDPs were chosen over SHAP (Shapley Additive Explanations) due to their computational efficiency and ease of interpretation. SHAP values are computationally expensive to calculate, particularly for large datasets or models with many features. Additionally, SHAP can be harder to interpret for non-technical audiences, as it provides complex, instance-level explanations that may not be as intuitive for broader analysis. In contrast, PDPs provide a global interpretation of the model’s behavior, showing how each feature influences the predicted outcome on average across the entire dataset.

Finally, stability testing was conducted by evaluating the model’s performance across different random seeds and data splits. This step confirmed the robustness of the model under varying conditions, further validating its reliability for real-world applications. The combination of cross-validation, hyperparameter tuning, feature importance analysis, and stability testing ensured that the ensemble-based approach was both accurate and interpretable, meeting the study’s objectives.

## 3 Results

### 3.1 Statistical analysis

Table 2 presents the demographic characteristics, distributions, and odds ratios (ORs) comparing participants who agree that food delivery causes dietary disruption to those who remain neutral, using the latter group as the reference. The results showed significant variations in perceptions of dietary disruption caused by food delivery. Lebanon and Bahrain indicated a stronger perception of dietary disruption, with the highest agreement rates at 35.2%, compared to other Arab countries. Additionally, Kuwait, with 30.3% agreement, Egypt, with 22.3% agreement, and Qatar, at 25.5%, also showed relatively lower agreement rates compared to countries like Saudi Arabia (33.6%) and the Emirates (32.8%).

**Table 2:** Demographic characteristics, distribution, univariate analysis (*F*-test) and odds ratios for participants agreeing that food delivery causes dietary disruption, using neutral participants as the control group.

Features	Agree *n* (%)	Neutral *n* (%)	Total group *n* (%)	F (*p*-value)	Odd ratio 95% CI
Area of residency					
Bahrain	191 (35.2)	351 (64.8)	542		
Egypt	257 (22.3)	898 (77.7)	1,155		
Emirates	90 (32.8)	184 (67.2)	274		
Jordan	215 (28)	552 (72)	767		
Kuwait	157 (30.3)	362 (69.7)	519	1.042 (0.207)	0.99 (0.972,1.008)
Lebanon	692 (35.2)	1,276 (64.8)	1968		
Oman	53 (16.9)	261 (83.1)	314		
Palestine	35 (28.2)	89 (71.8)	124		
Qatar	49 (25.5)	143 (74.5)	192		
Saudi Arabia	257 (33.6)	507 (66.4)	764		
Age group					
18–30	2,905 (62.8)	1,523 (76.3)	4,428 (66.9)	18.78 (<0.001)	1.45 (1.231,1.707)**
31–60	1718 (37.2)	473 (23.7)	2,191 (33.1)		
Gender					
Male	1,042 (22.5)	560 (28.1)	1,602 (24.2)	37.65 (<0.001)	1.5 (1.32,1.705)**
Female	3,581 (77.5)	1,436 (71.9)	5,017 (75.8)		
Education level					
≤ High school	1,083 (23.4)	567 (28.4)	1,650 (24.9)		1.097 (0.99,1.205)*
Bachelor degree	2,794 (60.4)	1,194 (59.8)	3,988 (60.3)	3.41 (0.064)	
≥ Master degree	746 (16.1)	235 (11.8)	981 (14.8)		
Employment					
No	2,433 (52.6)	1,197 (60.0)	3,630 (54.8)	3.262 (0.071)	1.122 (0.99,1.273)*
Yes	2,190 (47.4)	799 (40.0)	2,989 (45.2)		
Marital status					
Single	2,776 (60.0)	1,448 (72.5)	2,989 (45.2)	7.195 (0.007)	1.229 (1.054,1.433)*
Married	1847 (40.0)	548 (27.5)	4,224 (63.8)		
BMI					
Normal	1869 (40.4)	958 (48.0)	2,827 (42.7)		1.120 (1.065,1.177)**
Underweight	538 (11.6)	267 (13.4)	805 (12.2)	18.08 (<0.001)	
Overweight	1,397 (30.2)	551 (27.6)	1948 (29.4)		
Obese	819 (17.7)	220 (11.0)	1,039 (15.7)		

* 0.01 < *p*-value< 0.1; ** *p*-value<0.001.

The most statistically significant features influencing agreement were age group, gender, and BMI. Participants aged 18–30 were significantly more likely to agree, with an OR of 1.45 (95% CI: 1.231–1.707, *p* < 0.001), compared to those aged 31–60. Gender also played a key role, with males showing a higher likelihood of agreement (OR: 1.5, 95% CI: 1.32–1.705, *p* < 0.001). BMI demonstrated notable variation, with individuals in the obese category (OR: 1.120, 95% CI: 1.065–1.177, *p* < 0.001) being more likely to agree than those with a normal BMI.

Other features, such as area of residency, education level, employment status, and marital status, showed moderate importance, with ORs close to 1 and less robust statistical significance. For instance, participants with a high school education or less and unemployed individuals had slightly elevated odds of agreement (*p*-values between 0.01 and 0.1).

Similarly, the distribution of participants who agreed with dietary disruption followed distinct patterns, with the majority from females (77.5%). These distributions highlight demographic tendencies in perceptions of food delivery’s impact on dietary habits, with younger, urbanized, and overweight or obese individuals being more affected.

### 3.2 ML analysis

In the ML analysis, age, residency, and gender groups were balanced to focus on a broader set of features that prioritize the capture of the underlying behaviors, perceptions, and service attributes influencing dietary patterns. Table 3 presents the performance metrics of four machine learning models (RF, XGBoost, CatBoost, and LightGBM), evaluating their sensitivity, precision, F1-score, ACC, AUC, and prediction time (in seconds). These metrics provide a comprehensive view of the models’ ability to correctly identify dietary disruption and their efficiency in terms of computational time.

**Table 3:** Performance metrics and prediction time for ML models in dietary disruption detection.

Model	Sensitivity	Precision	F1-Score	ACC	AUC	Time(s)
Random forest	0.9434	0.8482	0.8932	0.8437	0.9100	0.0153
XGBoost	0.9379	0.8400	0.8863	0.8331	0.8909	0.0130
CatBoost	0.9336	0.8418	0.8853	0.8323	0.8844	0.0040
LightGBM	0.9183	0.8388	0.8768	0.8210	0.8835	0.0030

Among the models, RF achieved the highest sensitivity (0.9434), closely followed by XGBoost (0.9379) and CatBoost (0.9336). Sensitivity measures the model’s ability to correctly identify true positives, and these results indicate that all three models are highly effective at detecting dietary disruption. In terms of precision, all models performed similarly, with RF and CatBoost slightly outperforming the others. However, when considering the F1-score, which balances sensitivity and precision, RF once again led with the highest score (0.8932).

**Figure 2:**
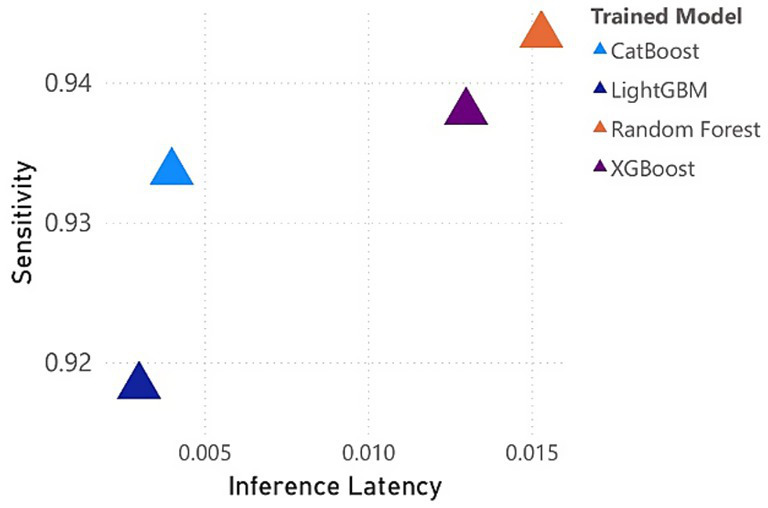
Trade-off between sensitivity and prediction time for machine learning models in detecting dietary disruption.

Interestingly, while RF excelled in predictive performance, it required slightly more prediction time (0.0153 s) compared to other models. LightGBM demonstrated the fastest prediction time (0.0030 s) while still maintaining a competitive F1-score and AUC, making it a practical choice for real-world applications where computational efficiency is critical, despite its slightly lower sensitivity compared to RF. The trade-off between prediction time and accuracy is clearly illustrated in Figure 2, where it is observed that while some models with marginally better sensitivity and precision require longer computation times. This trade-off highlights the importance of balancing model performance with computational efficiency, particularly for large-scale studies like this one, where timely insights are critical for informing interventions.

**Figure 3:**
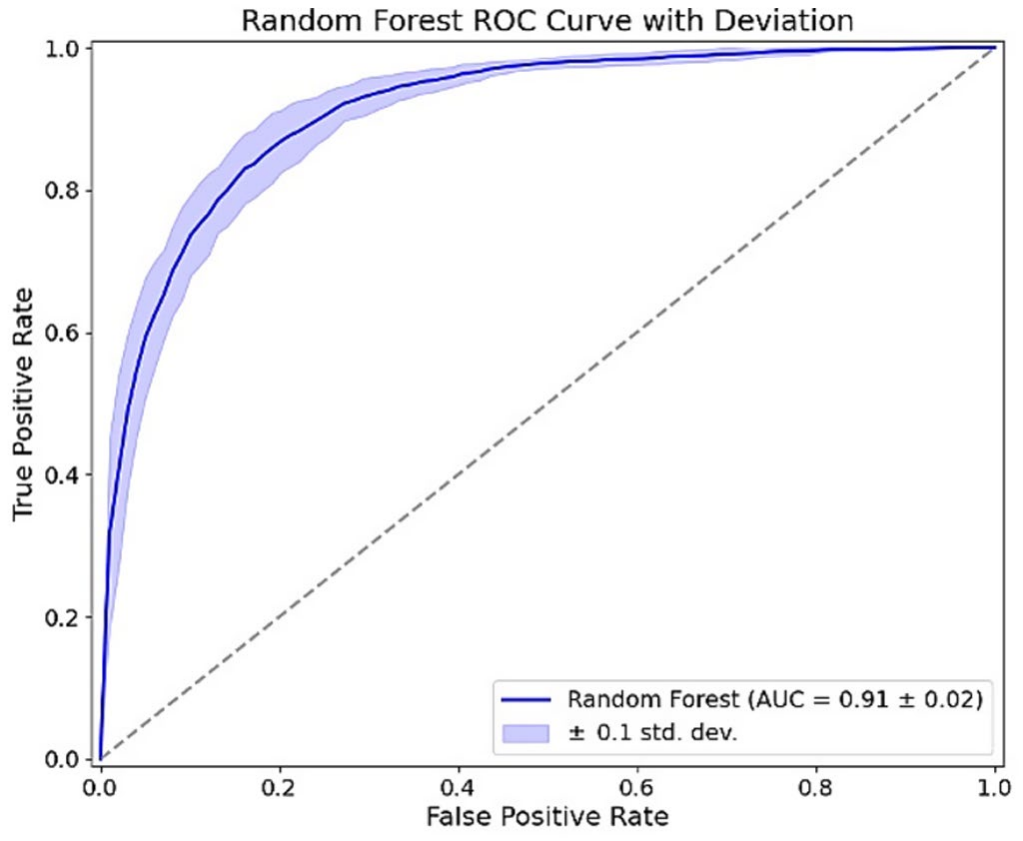
Predictive performance of the random forest model in identifying individuals at risk of dietary disruption, measured by the area under the ROC curve (AUC).

**Figure 4:**
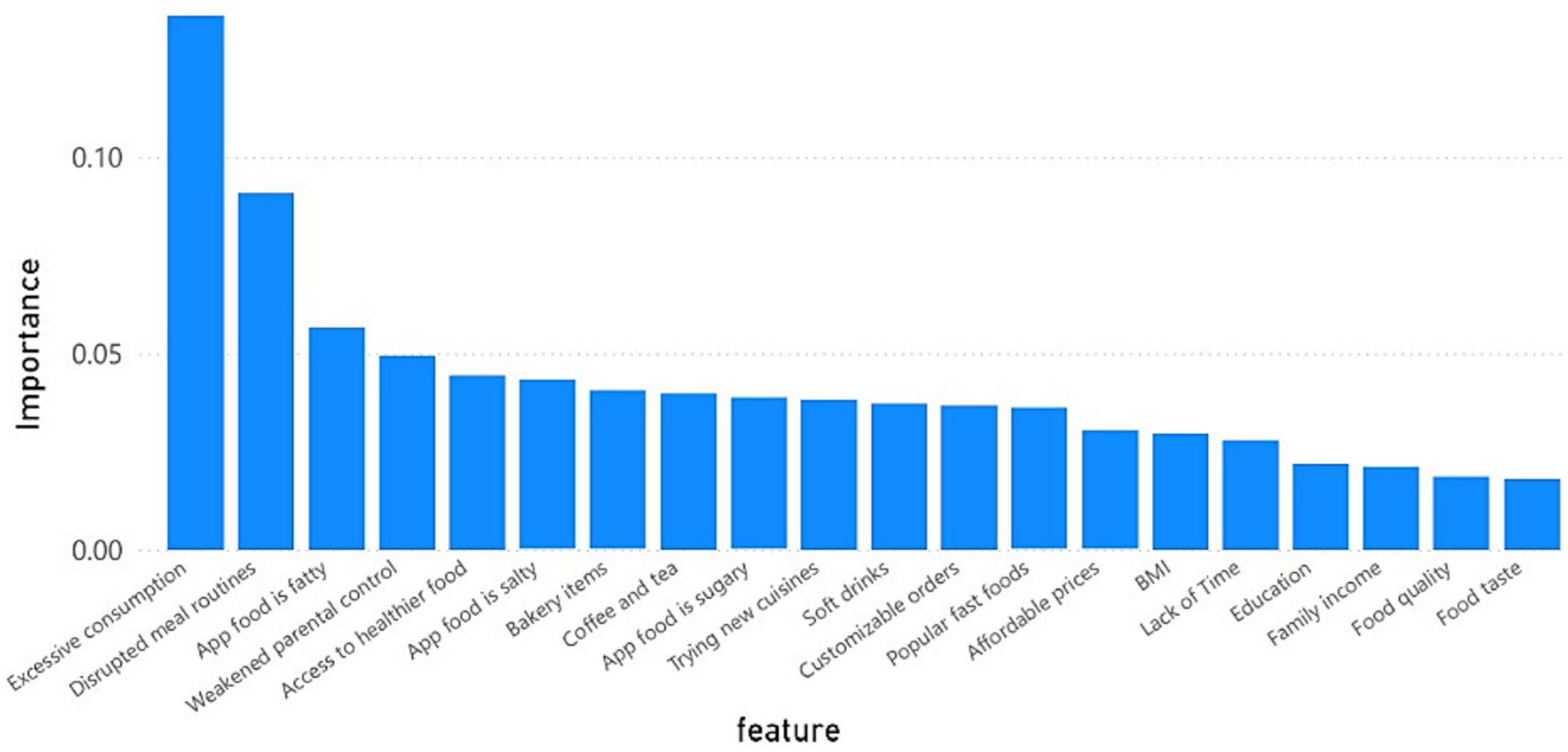
Key predictors of dietary disruption ranked by their influence in the random forest model.

The RF classifier exhibited strong predictive performance, as shown by the ROC curve in Figure 3. The model achieved an AUC of 0.91 ± 0.02, demonstrating excellent discriminative ability in identifying individuals at risk of dietary disruption. The shaded region represents the standard deviation across 10-fold cross-validation, as well as across different random seeds and data splits, demonstrating the model’s stability and consistent performance under varying conditions. This stable performance underscores the RF model’s reliability in identifying key factors contributing to dietary disruption Feature importance analysis from the RF model, illustrated in Figure 4, revealed the relative influence of various predictors on dietary disruption patterns. The features are ranked by their mean decrease in impurity, providing insights into which factors most strongly contribute to the model’s predictions.

Excessive consumption emerged as the most influential feature, with notably higher importance (>0.12) compared to other factors. This was followed by disrupted meal routines as the second most important predictor and appetites for fatty foods as the third. Weakened parental control and access to healthier food rounded out the top five features. While socioeconomic indicators such as education, family income, and food affordability showed relatively lower importance, their inclusion in the top 20 features suggests they still maintain meaningful predictive value in understanding dietary disruption patterns.

**Figure 5:**
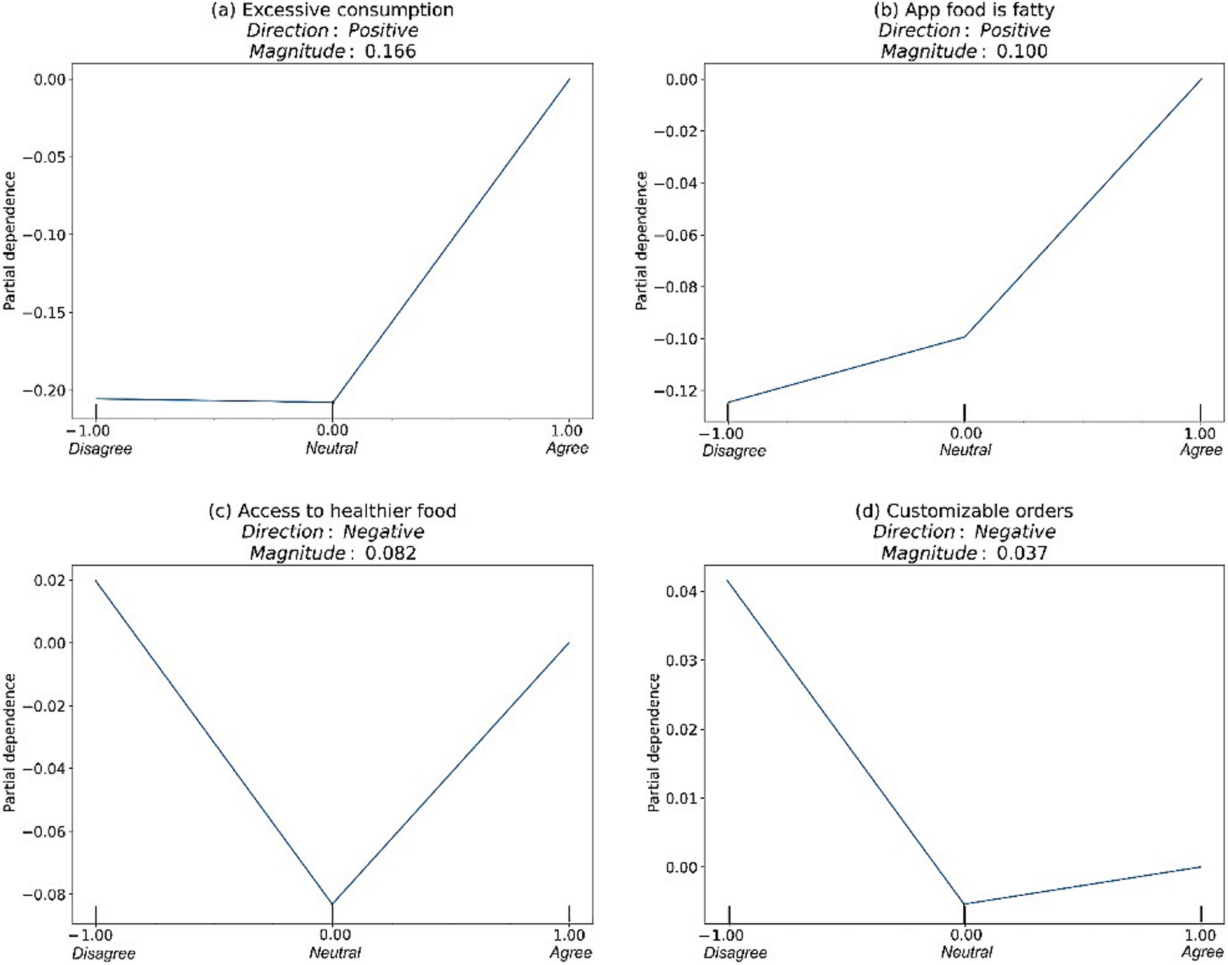
Marginal effects of key predictors on dietary disruption, as revealed by partial dependence plots (PDPs).

The partial dependency plots in Figure 5 reveal varying effects of four key features on dietary disruption. Excessive consumption emerged as the strongest predictor with a positive direction (magnitude = 0.166), indicating that participants who agreed that online food ordering leads to excessive consumption were more likely to experience dietary disruption. Similarly, the perception of app-delivered food being fatty showed a positive effect (magnitude = 0.100), suggesting that those who perceived delivery app food as high in fat content also reported greater dietary pattern disruption.

Interestingly, two features demonstrated negative effects. Participants who disagreed with having improved access to healthy options through delivery apps were more likely to report dietary disruption, as shown with the access to healthier food options feature (magnitude = −0.082). Similarly, the customizable orders feature (magnitude = −0.037) showed that those who disagreed with having order customization options were more prone to experiencing disrupted dietary patterns.

## 4 Discussion

The results showed significant demographic and behavioral patterns associated with dietary disruption linked to online food delivery (OFD) platforms. In comparison to previous research from other regions, our findings on age, gender, and BMI as key predictors are consistent, yet the magnitude of these effects appears amplified in the Arab context due to unique cultural and socio-economic factors (11). Lebanon and Bahrain exhibited the highest agreement rates (35.2%), indicating a stronger perception of OFD-induced dietary disruption in these regions. This could be due to Lebanon’s prolonged economic instability, shifting dietary patterns toward affordable fast-food options provided by OFDAs. In Bahrain, rapid economic growth, high disposable income, substantial expatriate populations, and increasing digital literacy have facilitated significant OFDA adoption. Coupled with the extensive presence of international food chains accessible through apps, this encourages shifts away from traditional home-cooked meals toward frequent consumption of fast foods, intensifying dietary disruption. In contrast, Oman showed the lowest agreement rate (16.9%), suggesting minimal perceived disruption. This finding aligns with a previous cross-sectional study in Oman, which reported that participants had fair to good control over online food ordering and found no direct correlation between OFDAs and obesity or overweight, despite their prevalence (42). This variation may reflect cultural, dietary, and OFD market differences across the Arab region, highlighting the need for further research on the specific factors contributing to greater dietary disruptions across different Arab countries.

The analysis identified age, gender, and BMI as key predictors of dietary disruption. Younger individuals (aged 18–30) were significantly more likely to perceive disruption (OR: 1.45, *p* < 0.001), potentially due to greater engagement with OFD platforms and more flexible eating habits. Males showed a higher likelihood of agreement (OR: 1.5, *p* < 0.001), which could be linked to gender-specific food consumption behaviors, including higher calorie intakes and preferences for convenience. This is consistent with previous findings that explored gender-based differences in opinions and practices regarding dietary behaviors. These studies showed that women were more likely to adhere to dietary guidelines and health recommendations, and their dietary choices, more often than those of men, corresponded to the principles of healthy nutrition (43–45). Obese individuals also demonstrated increased odds of perceiving dietary disruption (OR: 1.12, *p* < 0.001), supporting the hypothesis that OFD platforms may exacerbate unhealthy eating patterns in populations already at risk of poor dietary health.

Other demographic factors, such as residency, education, and employment status, exhibited moderate influence, with ORs close to 1. Participants with lower education levels or unemployed status showed slightly elevated odds of agreement (*p*-values between 0.01 and 0.1). Consistent with previous studies, individuals with lower levels of education tend to have less healthful diets (46, 47). Conscious food choices, therefore, must play an important role in mitigating the otherwise disruptive effects of OFDAs on health. The moderate effect of these elements aligns with literature suggesting that socioeconomic factors may modulate food access and dietary behaviors, though their effects may be less pronounced in the digital food environment (11, 15, 16).

Furthermore, socio-cultural factors, including religion, food preferences, education, and increased women’s employment, along with food subsidy policies in many Arab countries, which facilitate access to sugar, rice, wheat, and flour, largely affect food consumption patterns (48, 49). These subsidized staples, which are traditionally found in most Arab households, constitute the main ingredients for fast food, which could explain their relatively cheap price. The improved affordability of these unhealthy food items together with better accessibility through convenient, easy-to-use OFDAs could explain the increased occurrence of dietary disruptions. Accordingly, we highly advocate for food subsidy policies that also include healthy items like fruits and vegetables, to eliminate affordability as a negative determinant of the increased effect of OFDAs on food consumption habits and dietary choices. In addition to economic factors, cultural and religious dietary norms also significantly influence food choices through OFDAs. These include halal dietary restrictions, fasting practices during Ramadan, and social dining customs, all of which act as moderating factors in food selection. For instance, during Ramadan, OFDAs often market special meal packages for Iftar and Suhoor with the promise of convenience, thereby increasing access to non-traditional, calorie-dense fast-food options.

The machine learning (ML) analysis provided deeper insights into the behavioral and service-related determinants of dietary disruption. Among the models tested, Random Forest (RF) demonstrated the best predictive performance, achieving the highest sensitivity (0.9434) and F1-score (0.8932). This model’s robust performance highlights its suitability for identifying complex interactions among predictors. While LightGBM offered faster prediction times, its slightly lower sensitivity and F1-score showed the trade-off between computational efficiency and predictive accuracy.

The feature importance analysis from the RF model highlighted excessive consumption, disrupted meal routines, and preferences for fatty foods as the most influential predictors. These findings align with previous research indicating that OFD platforms tend to promote overconsumption and unhealthy food choices due to the availability and marketing of calorie-dense, nutrient-poor options (11, 15, 16). Excessive food consumption emerged as the strongest predictor dietary disruptions, emphasizing the behavioral risks associated with the convenience of OFD services. Designing targeted public health interventions that can mitigate excessive consumption patterns, such as digital nudges, pricing strategies, or educational campaigns, are essential. This aligns with (11) which documented how OFD availability has fundamentally altered traditional meal timing and frequency. Their study revealed that convenience and time-saving were primary motivators for OFD use, often leading to changes in established meal patterns and increased food consumption.

The identification of disrupted meal routines as the second most important predictor highlights the significant role that OFDAs play in altering traditional eating habits and regular meal schedules. Irregular eating patterns are associated with adverse health outcomes, including impaired metabolism, weight gain, and a higher risk of chronic diseases such as diabetes and cardiovascular disorders (11, 15, 16). Previous studies also found that OFD platforms often lead to irregular eating patterns due to increased frequency of food ordering (50).

Appetites for fatty foods emerged as the third most influential factor. OFD platforms often prioritize fast food and comfort meals due to their popularity and profitability, which reinforces unhealthy dietary choices. The frequent consumption of such foods has been linked to obesity, poor cardiovascular health, and other non-communicable diseases (11, 15, 16). This finding aligns with existing research demonstrating how the proliferation of unhealthy food options through delivery services exacerbates dietary imbalance and contributes to the global burden of diet-related diseases (11, 15, 16). Consistent with our findings, (51) also revealed how the digital food environment tends to promote less healthy dietary choices, particularly during periods of increased delivery service usage.

The inclusion of weakened parental control among the top five predictors emphasizes the challenges parents face in monitoring their children’s dietary choices. With children and adolescents increasingly exposed to digital platforms and their targeted marketing strategies, parental oversight is diminished. This shift may lead to a higher consumption of fast foods and sugary items, fostering unhealthy eating habits early in life. Additionally, many younger adults within this group (18–30 years) experience a greater degree of independence and reduced parental oversight, which may contribute to the observed weakened parental control over dietary choices.

On the other hand, while the presence of healthy food options on OFD platforms is a step forward, their impact is often diminished by the overwhelming dominance of less healthy alternatives. This suggests that improving not only the availability but also the promotion and affordability of healthier choices could help mitigate the adverse effects of OFD.

The negative relationship between access to healthy food options and dietary disruption provides an interesting counterpoint to existing literature. While (8) found that merely increasing healthy menu options did not significantly improve dietary choices, our results suggest that perceived access to healthy options may play a protective role against dietary disruption. This highlights the importance of not just availability but also visibility and promotion of healthier choices on OFD platforms. Further research should also consider psychological, social, and economic factors that drive food delivery behaviors, in order to design more targeted interventions.

The relatively lower importance of socioeconomic indicators in our model contrasts with traditional dietary research but aligns with recent findings by (52), which observed that while OFD platforms have increased food access across different socioeconomic groups in the UK, the predominance of unhealthy options remains a concern regardless of socioeconomic status. Their cross-sectional analysis demonstrated how the digital food environment might be reinforcing unhealthy eating patterns across all social strata. Accordingly, and while socio-cultural and economic factors are key disruptors, their effect is slightly less pronounced vis-à-vis the convenience factor and ease of access correlated with OFDAs. This suggests that while the latter’s adverse effect is not necessarily shrinking or increasingly becoming less tangible, the convenience element inherent to the design of OFDAs might diminish their outcome in comparison. In fact, elements of convenience - such as ease of access, time efficiency, and immediate gratification - are likely becoming the fundamental drivers of food choice, particularly in digital spaces. This raises new questions about how we can redesign these apps to retain their convenience while reducing its negative influence on dietary patterns.

In the same way that OFDAs can shape and reinforce consumer preferences over time, often leading to habitual overconsumption of unhealthy foods, recommendation algorithms promoting healthier options could be strategically integrated into these apps to influence dietary habits in a more positive direction (53, 54). Successful public health interventions in digital contexts, such as those integrating mobile health applications and targeted online campaigns, have shown promise in modifying dietary behaviors. Incorporating lessons from these interventions could inform strategies to counteract the adverse effects of OFD platforms (52).

These findings suggest that while OFD platforms have transformed food access, they have also introduced new challenges for maintaining healthy dietary patterns. The results highlight the need for targeted interventions that address behavioral triggers and leverage existing platform features to promote healthier choices.

## 5 Conclusion

The study demonstrates the significant impact of online food delivery (OFD) applications on dietary patterns in the Arab region, with excessive consumption, altered meal routines, and preferences for fatty foods emerging as the strongest predictors of dietary disruption. Weakened parental control and access to healthier food options further highlight the behavioral and environmental factors influencing these patterns. Demographic variations, particularly among younger individuals, males, and those with higher BMI, showed the vulnerability of specific groups to the adverse effects of OFD. Regional differences, with Lebanon and Bahrain reporting the highest disruption rates and Oman the lowest, reflect the influence of cultural and contextual factors. By addressing the influence of OFDAs on dietary behaviors through strategic public health and policy measures, these interventions can help shift OFDAs from disruptors of healthy eating to tools for improving nutrition in the region. A few of the measures we recommend include the use of gamification to encourage healthier food choices (55, 56), mandating nutritional labeling on OFDA platforms, advocating for the subsidization of healthful foods to promote affordability, redesigning OFDAs to maintain the convenience element while eliminating the adverse effects they may induce, and promoting better understanding of nutrition through targeted education programs to establish a culture of healthy eating habits.

### 5.1 Limitations and future work

This study provides valuable insights into the role OFDAs play in disrupting dietary patterns in the Arab region. Its key strengths lie in its large sample size (*n* = 7,370) and broad geographical coverage across 10 Arab countries. However, this study has several limitations. First, Convenience sampling could impact the external validity (generalizability) of the study’s conclusions, as participants might not accurately represent the broader population in terms of socioeconomic status, digital literacy, dietary preferences, or cultural diversity. This selection bias may lead to over- or underestimating the true impact of OFDAs on dietary patterns, especially since convenience samples often disproportionately include individuals who actively use technology or have stronger opinions regarding dietary habits. Second, online distribution through university networks and social media could introduce selection bias, favoring individuals with internet access and higher digital literacy. Third, self-reported data may be subject to recall and social desirability biases, potentially affecting response accuracy. Fourth, no pre-screening criteria were applied to ensure prior use of online food delivery applications (OFDAs), meaning responses may include individuals with little or no prior engagement, potentially influencing perceptions and reported behaviors. Finally, while efforts were made to ensure cultural relevance, some variations in interpretation of questions across countries may have influenced responses.

Future research will further explore the interplay between food safety and consumer ordering habits through OFD apps. Specifically, it will investigate how these behaviors influence the likelihood of encountering food safety risks and illness, providing actionable insights to improve food delivery practices.

## Data Availability

The raw data supporting the conclusions of this article will be made available by the authors, without undue reservation.
